# Supplementary material for: Characterization of the innate immune response to Streptococcus pneumoniae infection in zebrafish
Source: PLoS Genet. 2023 Jan 9;19(1):e1010586. doi: 10.1371/journal.pgen.1010586 (PMC9858863; doi:10.1371/journal.pgen.1010586)
Supplement: S1 Table — (PDF) [file pgen.1010586.s001.pdf]

S1 Table. The induced protein coding genes not previously reported in the context of immune response.

Gene symbol	Gene name	Ensembl gene ID	Fold change
**metabolic process**			
zgc:153968		ENSDARG00000061858	11.9
ctrb.3	*chymotrypsinogen B, tandem duplicate 3*	ENSDARG00000039730	5.0
CABZ01088134.1		ENSDARG00000033587	4.5
ela3l	*elastase 3 like*	ENSDARG00000007276	3.9
cela1.6	*chymotrypsin-like elastase family member 1 tandem duplicate 6*	ENSDARG00000017314	3.8
ttr	*transthyretin (prealbumin, amyloidosis type I)*	ENSDARG00000037191	3.7
ctrb1	*chymotrypsinogen B1*	ENSDARG00000090428	3.7
cyp7a1	*cytochrome P450, family 7, subfamily A, polypeptide 1*	ENSDARG00000069018	3.2
gls2a	*glutaminase 2a (liver, mitochondrial)*	ENSDARG00000069095	3.2
prss59.2	*serine protease 59, tandem duplicate 2*	ENSDARG00000073742	3.2
ctrl	*chymotrypsin-like*	ENSDARG00000068680	3.1
prss59.1	*serine protease 59, tandem duplicate 1*	ENSDARG00000079274	3.0
**other function**			
BX548011.1		ENSDARG00000103357	9.4
slc2a6	*solute carrier family 2 (facilitated glucose transporter), member 6*	ENSDARG00000058731	4.3
sult5a1	*sulfotransferase family 5A, member 1*	ENSDARG00000007769	3.8
slc23a3	*solute carrier family 23, member 3*	ENSDARG00000088891	3.8
odam	*odontogenic, ameloblast associated*	ENSDARG00000074476	3.4
pde6h	*phosphodiesterase 6H, cGMP-specific, cone,*	ENSDARG00000102558	3.3
slc37a4b	*solute carrier family 37, member 4b*	ENSDARG00000093531	3.2
**unknown function**			
CU539054.1		ENSDARG00000088292	8.2
sc:d217		ENSDARG00000079645	5.1
si:dkey-27h10.2		ENSDARG00000094485	4.7
si:ch1073-126c3.2		ENSDARG00000092858	4.4
si:ch73-263o4.3		ENSDARG00000096207	4.5
hpxb	*hemopexin b*	ENSDARG00000051912	4.3
si:ch211-194m7.3		ENSDARG00000074322	4.2
si:ch211-284o19.8		ENSDARG00000053836	4.1
BX908782.1		ENSDARG00000098957	4.0
si:dkey-102g19.3		ENSDARG00000086337	3.6
si:dkey-9c18.3		ENSDARG00000096579	3.4
zgc:173443		ENSDARG00000034403	3.4

tmem176l.2	ENSDARG00000089399	3.2

The table shows the fold change in expression in *S. pneumoniae* infected larvae compared to the KCl injected larvae at 18 hpi. The data comprise three biological replicates and the fold change was calculated using the DEseq2-tool. The table includes only the genes with a mean normalized read count of ≥20 after infection, and genes induced by at least 3.0-fold compared to KCl injected larvae.
