# Supplementary material for: Characterization of the innate immune response to Streptococcus pneumoniae infection in zebrafish
Source: PLoS Genet. 2023 Jan 9;19(1):e1010586. doi: 10.1371/journal.pgen.1010586 (PMC9858863; doi:10.1371/journal.pgen.1010586)
Supplement: S2 Table — (PDF) [file pgen.1010586.s002.pdf]

S2 Table. Downregulated protein coding genes in pneumococcal infection.

Gene symbol	Gene name	Ensembl gene ID	Fold change
**reproduction and development**			
zp3f.1	*zona pellucida glycoprotein 3f, tandem duplicate 1*	ENSDARG00000038720	-172.5
zp3a.1	*zona pellucida glycoprotein 3a, tandem duplicate 1*	ENSDARG00000042129	-67.5
zp3.2	*zona pellucida glycoprotein 3, tandem duplicate 2*	ENSDARG00000090768	-50.9
zp2.2	*zona pellucida glycoprotein 2, tandem duplicate 2*	ENSDARG00000105346	-24.0
zp3e	*zona pellucida glycoprotein 3e*	ENSDARG00000016908	-18.0
zpax1	*Zona pellucida protein AX 1*	ENSDARG00000069251	-13.3
zp3b	*zona pellucida glycoprotein 3b*	ENSDARG00000039828	-3.5
zp3a.2	*zona pellucida glycoprotein 3a, tandem duplicate 2*	ENSDARG00000042130	-3.2
**metabolic process**			
he1.1	*hatching enzyme 1, tandem duplicate 1*	ENSDARG00000023656	-4.4
**other function**			
hbba1	*hemoglobin, beta adult 1*	ENSDARG00000089087	-87.0
hbba1	*hemoglobin, beta adult 1*	ENSDARG00000097238	-55.1
tmsb5	*thymosin beta 5*	ENSDARG00000093886	-7.2
cldnd	*claudin d*	ENSDARG00000006580	-5.3
cldng	*claudin g*	ENSDARG00000003701	-5.1
h2af1al	*H2A histone family member 1a like*	ENSDARG00000098739	-4.8
**unknown function**			
zgc:173856		ENSDARG00000045424	-259.2
si:dkey-90l23.1		ENSDARG00000092057	-202.9
si:dkey-241l7.4		ENSDARG00000089478	-200.6
CABZ01059627.2		ENSDARG00000079681	-197.6
si:ch1073-75o15.3		ENSDARG00000095741	-141.1
si:ch211-250e5.16		ENSDARG00000078457	-124.4
CABZ01059627.1		ENSDARG00000078394	-65.6
wu:fi42e03		ENSDARG00000070709	-47.5
si:dkey-241l7.5		ENSDARG00000088989	-41.6
zgc:173837		ENSDARG00000076949	-40.4
zgc:171776		ENSDARG00000032156	-40.2
si:ch211-5k11.8		ENSDARG00000079078	-23.8
si:ch211-226h8.14		ENSDARG00000088713	-22.9
si:ch211-12h2.8		ENSDARG00000088923	-22.8
zgc:111868		ENSDARG00000099060	-21.4
zgc:171781		ENSDARG00000086247	-19.8
BX548000.1		ENSDARG00000079546	-16.1
si:dkey-240e12.6		ENSDARG00000088932	-3.1

The table shows the fold change in expression in *S. pneumoniae* infected larvae compared to the KCl injected larvae at 18 hpi. The data comprise three biological replicates and the fold change was

calculated using the DEseq2-tool. Only the genes with a mean normalized read count of ≥20 in KCl injected controls, and whose expression was reduced by at least 3.0-fold, are listed.
