# Supplementary material for: Characterization of the innate immune response to Streptococcus pneumoniae infection in zebrafish
Source: PLoS Genet. 2023 Jan 9;19(1):e1010586. doi: 10.1371/journal.pgen.1010586 (PMC9858863; doi:10.1371/journal.pgen.1010586)
Supplement: S3 Table — (PDF) [file pgen.1010586.s003.pdf]

S3 Table. Non-coding RNAs upregulated in pneumococcal infection.

Gene symbol	Biotype	Ensembl gene ID	Fold change
BX539325.1	processed transcript	ENSDARG00000095909	18.9
5S_rRNA	rRNA	ENSDARG00000101173	14.6
CABZ01059413.1	lincRNA	ENSDARG00000108218	12.4
SNORD52	snoRNA	ENSDARG00000084582	10.6
dre-mir-124-3	miRNA	ENSDARG00000081423	7.3
CABZ01027551.2	lincRNA	ENSDARG00000108463	6.5
CU326347.1	lincRNA	ENSDARG00000107705	5.3
BX649485.2	lincRNA	ENSDARG00000095801	5.0
SNORA7	snoRNA	ENSDARG00000082419	5.0
CR933734.2	processed transcript	ENSDARG00000093974	4.9
BX649485.4	lincRNA	ENSDARG00000095820	4.6
SNORA63	snoRNA	ENSDARG00000082928	4.6
CABZ01109079.1	lincRNA	ENSDARG00000108455	4.3
CR753876.1	lincRNA	ENSDARG00000097137	4.3
dre-mir-181a-1	miRNA	ENSDARG00000083193	4.1
CR855311.1	processed transcript	ENSDARG00000090352	3.9
SNORA65	snoRNA	ENSDARG00000083937	3.9
CABZ01056516.2	lincRNA	ENSDARG00000108320	3.7
CABZ01017833.1	lincRNA	ENSDARG00000105915	3.6
BX649307.1	antisense RNA	ENSDARG00000096046	3.1

The table shows the fold change in expression in *S. pneumoniae* infected larvae compared to the KCl injected larvae at 18 hpi. The data comprise three biological replicates and the fold change was calculated using the DEseq2-tool. The table includes only the genes with a mean normalized read count of ≥20 after infection, and genes induced by at least 3.0-fold compared to KCl injected larvae.
