# Supplementary material for: Characterization of the innate immune response to Streptococcus pneumoniae infection in zebrafish
Source: PLoS Genet. 2023 Jan 9;19(1):e1010586. doi: 10.1371/journal.pgen.1010586 (PMC9858863; doi:10.1371/journal.pgen.1010586)
Supplement: S4 Table — (PDF) [file pgen.1010586.s004.pdf]

S4 Table. Downregulated non-coding RNAs in pneumococcal infection.

Gene symbol	Biotype	Ensembl gene ID	Fold change
si:ch211-199m8.2	processed_transcript	ENSDARG00000099044	-17.4
SNORA65	snoRNA	ENSDARG00000083894	-9.5
U6	snRNA	ENSDARG00000104896	-6.7
dre-mir-130b	miRNA	ENSDARG00000080347	-6.3
AC024175.23	Mt_tRNA	ENSDARG00000083480	-6.2
5S_rRNA	rRNA	ENSDARG00000098086	-5.9
dre-mir-206-1	miRNA	ENSDARG00000082396	-4.8
CABZ01075924.1	lincRNA	ENSDARG00000107951	-4.6
CR318588.1	processed_transcript	ENSDARG00000092191	-4.5
SNORD35	snoRNA	ENSDARG00000080207	-4.4
dre-mir-124-2	miRNA	ENSDARG00000081311	-4.1
5S_rRNA	rRNA	ENSDARG00000106401	-3.9
dre-mir-181b-2	miRNA	ENSDARG00000081534	-3.5
BX323038.1	antisense_RNA	ENSDARG00000092791	-3.5
CABZ01040931.2	lincRNA	ENSDARG00000108685	-3.4
SNORA35	snoRNA	ENSDARG00000080450	-3.1
CABZ01085281.1	lincRNA	ENSDARG00000106696	-3.0
snoU85	snoRNA	ENSDARG00000104320	-3.0

The table shows the fold change in expression in *S. pneumoniae* infected larvae compared to the KCl injected larvae. The data comprise three biological replicates and the fold change was calculated using the DEseq2-tool. Only the genes with a mean normalized read count of ≥20 in KCl injected controls, and whose expression was reduced by at least 3.0-fold are listed.
