# Supplementary material for: Characterization of the innate immune response to Streptococcus pneumoniae infection in zebrafish
Source: PLoS Genet. 2023 Jan 9;19(1):e1010586. doi: 10.1371/journal.pgen.1010586 (PMC9858863; doi:10.1371/journal.pgen.1010586)
Supplement: S5 Table — (PDF) [file pgen.1010586.s005.pdf]

S5 Table. Downregulated protein coding genes in mutant94 larvae.

Gene symbol	Gene name	Ensembl gene ID	Fold Change
**Immune response**			
crp2	*C-reactive protein 2*	ENSDARG00000056498	-83.2
chia.1	*chitinase, acidic.1*	ENSDARG00000100635	-6.0
ly6m2	*lymphocyte antigen 6 family member M2*	ENSDARG00000104775	-5.5
chia.2	*chitinase, acidic.2*	ENSDARG00000099185	-4.2
**metabolic process**			
cyp7a1	*cytochrome P450, family 7, subfamily A, polypeptide 1*	ENSDARG00000069018	-7.1
ctrb.3	*chymotrypsinogen B, tandem duplicate 3*	ENSDARG00000039730	-3.5
**unknown function**			
BX548011.1		ENSDARG00000103357	-43.2
si:dkey-9c18.3		ENSDARG00000096579	-28.6
zgc:173443		ENSDARG00000034403	-3.8

The table shows the fold change in expression of the pneumococcus-responsive genes in *S. pneumoniae* infected mutant94 larvae compared to the infected AB larvae. The data comprise three biological replicates and the fold change was calculated with the DEseq2-tool. Only the genes with a mean normalized read count of ≥20 in AB larvae, and whose expression was reduced by at least 3.0-fold in mutants compared to AB are listed.
