# Supplementary material for: Characterization of the innate immune response to Streptococcus pneumoniae infection in zebrafish
Source: PLoS Genet. 2023 Jan 9;19(1):e1010586. doi: 10.1371/journal.pgen.1010586 (PMC9858863; doi:10.1371/journal.pgen.1010586)
Supplement: S6 Table — (PDF) [file pgen.1010586.s006.pdf]

S6 Table. Downregulated non-coding RNAs in mutant94 larvae.

Gene symbol	Biotype	Ensembl gene ID	Fold change
CABZ01027551.2	lincRNA	ENSDARG00000108463	-11.8
CR933734.2	processed_transcript	ENSDARG00000093974	-4.4
SNORD52	snoRNA	ENSDARG00000084582	-3.5
dre-mir-181a-1	miRNA	ENSDARG00000083193	-3.2
SNORA7	snoRNA	ENSDARG00000082419	-3.1
dre-mir-124-3	miRNA	ENSDARG00000081423	-3.0

The table shows the fold change in expression of the pneumococcus-responsive non-coding RNAs in *S. pneumoniae* infected mutant94 larvae compared to the infected AB larvae. The data comprise three biological replicates and the fold change was calculated using the DEseq2-tool. Only the genes with a mean read count of ≥20 in infected AB larvae, and whose expression was reduced by at least 3.0-fold in mutants compared to AB are listed.
