# Supplementary material for: Characterization of the innate immune response to Streptococcus pneumoniae infection in zebrafish
Source: PLoS Genet. 2023 Jan 9;19(1):e1010586. doi: 10.1371/journal.pgen.1010586 (PMC9858863; doi:10.1371/journal.pgen.1010586)
Supplement: S7 Table — (PDF) [file pgen.1010586.s007.pdf]

S7 Table. Primer sequences

Target gene	Purpose	Ensembl Gene ID	Forward 5'-->3'	Reverse 5'-->3'	Reference
crp2-1	genotyping/target site amplification and sequencing	ENSDARG00000056498	ATCTCAGTGGTAAAGTGCTTCAG	ATGGTGTCCCAGTCAAACAC	
crp2-2	genotyping/target site amplification and sequencing	ENSDARG00000056462	CCAATCGTCAGTTTTTCAGCA	CGAGGAGGTCAGGTTTAACTGAA	
crp3	genotyping/target site amplification and sequencing	ENSDARG00000042613	CACAGTTTAATGCAACACAGAGC	CAACATTTAGCATTGGTCCATGT	
eef1a1l1	qPCR	ENSDARG00000020850	CTGGAGGCCAGCTCAAACAT	ATCAAGAAGAGTAGTACCGCTAGCATTAC	[1]
il1b	qPCR	ENSDARG00000098700	TGGACTTCGCAGCACAAAATG	GTTCACTTCACGCTCTTGGATG	[2]
il6	qPCR	ENSDARG00000102318	TCAACTTCTCCAGCGTGATG	TCTTTCCCTCTTTTCCTCCTG	[3]
tnfa	qPCR	ENSDARG00000009511	GGGCAATCAACAAGATGGAAG	GCAGGTGATGTGCAAAGACAC	[4]
crp2-1	qPCR, Figure S1	ENSDARG00000056498	CAAGATGTTGGTTGTATTTCTC	CTCAGGGGTTTTTGAGGAGTCAGT	
crp2-1	qPCR, Figure 5	ENSDARG00000056498	GACGTCGCAGTTTGCAC	CCACAAAGCTCTGATCTACATC	
crp2-2	qPCR	ENSDARG00000056462	GCTGAAGTGGGTCTCAGTG	GTAGGCGAACAGGATGATC	
crp3	qPCR	ENSDARG00000042613	GAAGTGGGTCTCGGTG	GTAGGCGAACAGGATGAC	

References

1 Tang R, Dodd A, Lai D, McNabb WC, Love DR. Validation of zebrafish (*Danio rerio*) reference genes for quantitative real-time RT-PCR normalization. Acta Biochim Biophys Sin. 2007; 39: 384-390.

2 Pressley ME, Phelan PE, Witten PE, Mellon MT, Kim CH. Pathogenesis and inflammatory response to *Edwardsiella tarda* infection in the zebrafish. Dev Comp Immunol. 2005; 29: 501-513.

3 Patterson H, Saralahti A, Parikka M, Dramsi S, Trieu-Cuot P, Poyart C, et al. Adult zebrafish model of bacterial meningitis in *Streptococcus agalactiae* infection. Dev Comp Immunol. 2012; 38: 447-455.

4 Myllymäki H, Niskanen M, Luukinen H, Parikka M, Rämet M. Identification of protective postexposure mycobacterial vaccine antigens using an immunosuppression-based reactivation model in the zebrafish. Dis Model Mech. 2018;11(3):dmm033175.
