# Supplementary material for: Characterization of the innate immune response to Streptococcus pneumoniae infection in zebrafish
Source: PLoS Genet. 2023 Jan 9;19(1):e1010586. doi: 10.1371/journal.pgen.1010586 (PMC9858863; doi:10.1371/journal.pgen.1010586)
Supplement: S1 Fig — (PDF) [file pgen.1010586.s008.pdf]

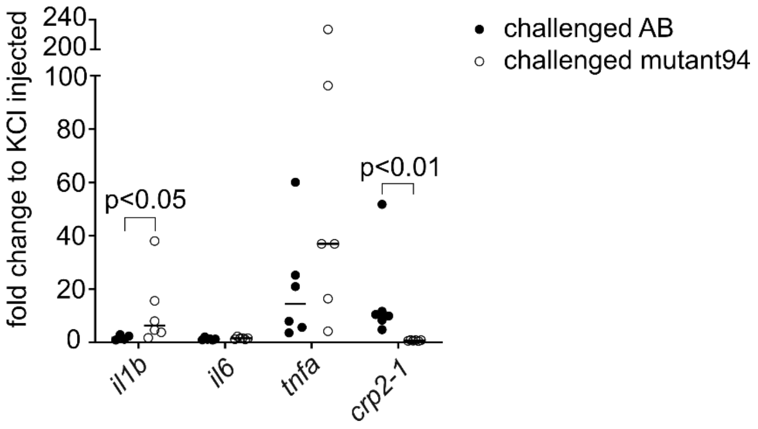

S1 Fig. Relative expression of *il1b, il6, tnfa, and crp2-1* in AB and mutant94 larvae.

A dot/circle represents relative gene expression in a single pool of wild type larvae (challenged AB) and mutant94 larvae (challenged mutant94) infected with ~500 cfu of *S. pneumoniae* compared to the AB larvae injected with KCl. The line depicts the median expression level. The gene expression levels were measured from the pools of five larvae at 18 hpi and the expression of studied genes were normalized to the expression of *eef1a1l1*. $2^{-\Delta\Delta Ct}$ method was used to calculate the differential expression and the statistical comparisons of difference were calculated with two-tailed Mann-Whitney test.
