# Supplementary material for: Characterization of the innate immune response to Streptococcus pneumoniae infection in zebrafish
Source: PLoS Genet. 2023 Jan 9;19(1):e1010586. doi: 10.1371/journal.pgen.1010586 (PMC9858863; doi:10.1371/journal.pgen.1010586)
Supplement: S2 Fig — (PDF) [file pgen.1010586.s009.pdf]

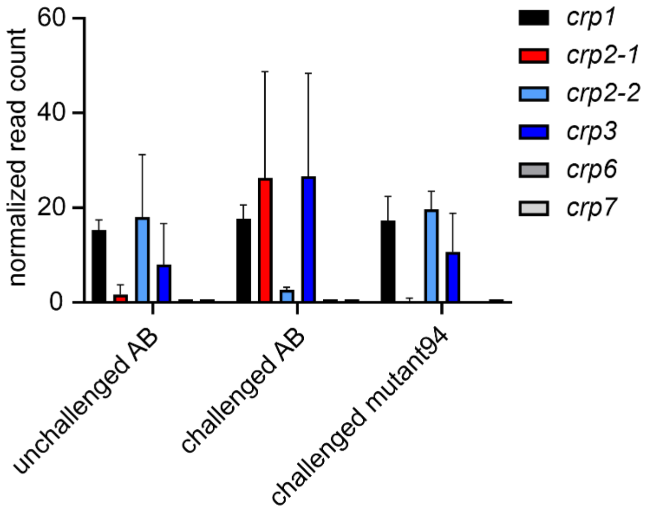

S2 Fig. Expression of zebrafish Crp-encoding genes in AB and mutant94 larvae at 18 hours post pneumococcal challenge

The figure shows the mean normalized read counts for *crp1, crp2-1, crp2-2, crp3, crp6*, and *crp7* in AB larvae injected with KCl (unchallenged AB), AB larvae infected with ~500 cfu of *S. pneumoniae* (challenged AB), and mutant94 larvae infected with ~500 cfu of *S. pneumoniae* (challenged mutant94) at 18 hpi. The data consist of three biological replicates (10 larvae in each) and the error bars represent standard deviation. The differential expressions did not reach statistical significance.
