# Supplementary material for: Characterization of the innate immune response to Streptococcus pneumoniae infection in zebrafish
Source: PLoS Genet. 2023 Jan 9;19(1):e1010586. doi: 10.1371/journal.pgen.1010586 (PMC9858863; doi:10.1371/journal.pgen.1010586)
Supplement: S3 Fig — (PDF) [file pgen.1010586.s010.pdf]

A

```
reference_crp2-1  AAGTTTACAGGAAAGGTTTCTCCATTCGTCCTGGCGGCACCGTCGTGCTCGGACAAGACC        420
homozygous_1      -----------------------------CCTGGCGGCACCGTCCTGCTCGGACAAGACC        31
homozygous_2      --------------------------CGTCCTGGCGGCACCGTCGTGCTCGGACAAGACC        34
homozygous_3      --------------------------CGTCCTGGCGGCACCGTCCTGCTCGGACAAGACC        34
homozygous_4      ---------------------------GTCCTGGCGGCACCGTCCTGCTCGGACAAGACC        33
                                               *************** ***************

reference_crp2-1  CTGATTCATATCTAGGTTCCTTTGATGTAGATCAGAGCTTTGTGGGAGAAATTGCAAATC        480
homozygous_1      CTGATTCATATGTAGGTTCTTTTGATGTAGATCAGAGCTTTGTG----------------        75
homozygous_2      CTGATTCATATCTAGGTTCTTTTGAT----------------------------------        60
homozygous_3      CTGATTCATATGTAGGTTCTTTTGATGTAGATCAGAGCTTTGTGGA--------------        80
homozygous_4      CTGATTCATATGTAGGTTCTTTTGATKTAGATCAGAGCTTTGTGGA--------------        79
                  ***********.******* ******
```

B

```
reference_crp2-2  TACTCCATTCGTCCTGGCGGCACCGTCCTGCTCGGACAAGACCCGGATTCATATGTAGGT        660
homozygous_1      ------------CCTGGCGGCACCGTCCTGCTCGGACAAGACCCTGATTCATATGTAGGT        48
homozygous_2      ---------CGTCCTGGCGGCACCGTCGTGCTCGGACAAGACCCTGATTCATATCTAGGT        51
homozygous_3      ---------CGTCCTGGCGGCACCGTCCTGCTCGGACAAGACCCTGATTCATATGTAGGT        51
homozygous_4      ----------GTCCTGGCGGCACCGTCCTGCTCGGACAAGACCCTGATTCATATGTAGGT        50
                              ***************.**************** *********.*****

reference_crp2-2  TCCTTTAATGCAGCTCAGAGCTTTGTGGGAGAAATTACGGATCTGCAAATGTGGGACTAT        720
homozygous_1      TCTTTTGATGTAGATCAGAGCTTTGTG---------------------------------        75
homozygous_2      TCTTTTGAT---------------------------------------------------        60
homozygous_3      TCTTTTGATGTAGATCAGAGCTTTGTGGA-------------------------------        80
homozygous_4      TCTTTTGATKTAGATCAGAGCTTTGTGGA-------------------------------        79
                  ** ***.**
```

C

```
reference_crp3    ACGTCGTAGTTCGTACCAAGTGTACAGGGAAAGGTTACTCTATTCKTTCTGGCGGCACTG        600
homozygous_1      -----------------------------------------------CCTGGCGGCACCG        13
homozygous_2      --------------------------------------------CGTCCTGGCGGCACCG        16
homozygous_3      --------------------------------------------CGTCCTGGCGGCACCG        16
homozygous_4      ---------------------------------------------GTCCTGGCGGCACCG        15
                                                                ********** *

reference_crp3    TCCTGCTCGGACAAGACCCTGATTCATATGTAGGTTCCTTTGATGTAGATCAGAGTTTTG        660
homozygous_1      TCCTGCTCGGACAAGACCCTGATTCATATGTAGGTTCTTTTGATGTAGATCAGAGCTTTG        73
homozygous_2      TCGTGCTCGGACAAGACCCTGATTCATATCTAGGTTCTTTTGAT----------------        60
homozygous_3      TCCTGCTCGGACAAGACCCTGATTCATATGTAGGTTCTTTTGATGTAGATCAGAGCTTTG        76
homozygous_4      TCCTGCTCGGACAAGACCCTGATTCATATGTAGGTTCTTTTGATKTAGATCAGAGCTTTG        75
                  **.**************************.******* ******
```

S3 Fig. Alignment of qPCR product sequences from homozygous *crp2*$^{tpu6/tpu6}$ mutants with *crp2-1*, *crp2-2* and *crp3* reference sequences.

The qPCR products obtained with *crp2-1* specific primers from *S. pneumoniae* challenged homozygous larvae (homozygous 1-4) were aligned with the A) *crp2-1*, B) *crp2-2* and C) *crp3* reference sequences with Clustal omega multiple sequence alignment tool. Reference sequences were obtained by Sanger sequencing of multiple wild type AB larvae. Bases differing from the reference sequence are highlighted in grey.
