# Supplementary material for: Characterization of the innate immune response to Streptococcus pneumoniae infection in zebrafish
Source: PLoS Genet. 2023 Jan 9;19(1):e1010586. doi: 10.1371/journal.pgen.1010586 (PMC9858863; doi:10.1371/journal.pgen.1010586)
Supplement: Attachment — Submitted filename: Response to Reviewers.pdf [file pgen.1010586.s012.pdf]

Response letter Tampere 9th of November 2022

Dear Editor and Reviewers,

We sincerely thank you for your valuable comments on our manuscript "Characterization of the innate immune response to *Streptococcus pneumoniae* infection in zebrafish" (PGENETICS-D-22-00563). Please find below the point-by-point responses to reviewer's comments and a summary of the experiments and data added to the revised article. Reviewers' comments and questions are shown in black and bolded, our responses are in black and the added and modified parts of the text are in blue. The line numbers presented in this Response letter correspond to the line numbers in the file named as "Manuscript". The modifications in the separate file "Revised Article with Changes Highlighted" are marked by the "Track Changes" option.

Comments from the Reviewer 1

This study leverages a zebrafish larval model of Streptococcus pnuemoniae infection to identify genes involved in innate immune response to sepsis. The authors use transcriptomics to characterize the acute inflammatory response to Spn in larvae, demonstrating that immune pathways activated in zebrafish are similar to the immune pathways that are well known to be activated in human and mammalian infections – such as complement factors. The authors then perform a forward genetic screen to identify mutants with increased susceptibility to Spn infection and identify a hit in the gene encoding C-reactive protein (CRP). CRP is well known clinically as an important sign of acute inflammation, therefore demonstrating consistency and conservation of the response between zebrafish, a cold-blooded vertebrate and humans. The experiments are well designed and provides an extremely useful foundation to validate this underutilized model. However, ultimately, the putative role for CRP in defense against Spn was not genetically validated, as the targeted CRISPR mutant did not recapitulate the severe phenotype of the mutant identified in their screen. Thus as it stands, this work has the potential to be useful to the field, but the conclusions of the role of Crp2-1 in zebrafish is not sufficiently supported by experimental evidence – <u>this is a major concern that should be addressed before publication.</u> At a minimum, the authors should provide genetic evidence of the factor responsible for susceptibility in their line (perhaps *crp3* or *crp2-2*, or a second gene). Generating double knockout lines would be the most compelling but is a significant amount or work / time to complete. Instead, the authors may be able to address these concerns with morpholino knockdowns. Additionally, this paper would be improved with experimental mechanisms or validation beyond transcription (i.e. protein level or functional killing against S. pneumoniae) though this is not a critical for publication in my opinion.

Major concerns

1) It is disappointing that after all the work making the various lines, the phenotype is not recapitulated in the CRISPR deletion of *crp2-1*. Based on the challenges with qPCR, the possible

compensatory effect of the other crp's as the authors point out, or the possibility that the mutant94 had another mutation, this is a critical concern that should be addressed. As it stands, the title of this section (line 302: Knockout of *crp2-1* renders zebrafish larvae more susceptible to systemic pneumococcal infection) is not supported by their data. Although there is technically a very small but significant difference in survival, this looks to be something that's only possible with very high n and only when analyzed at the late time point. It certainly does not recapitulate the phenotype seen in the mutant94.

In lieu of making a double knockout line, the authors might try using morpholinos to knock down crp3 in the crp2-1$^{tpu6/tpu6}$ line. If that is not able to recapitulate the phenotype, the authors might try knocking down the other differentially expressed genes (BX548011.1 (43-fold decrease in expression) and si:dkey-9c18.3 (29-fold decrease in expression). Alternatively, they might sequence the genome of the mutant94 line to see if it really was inserted in crp2-1, or perhaps was inserted into a master regulator that results in differential crp2-1 expression.

We agree with the reviewer's comment that the phenotype of *crp2-1* KO mutant certainly does not copy the phenotype of mutant94, but instead shows modest decrease in survival compared to wild type line. Therefore, we do agree that additional data are needed to make the manuscript stronger.

According to the reviewer's suggestion, we designed morpholino oligonucleotides for the knockdown of *crp3*, *crp2-2* and *BX548011.1* in *crp2-1* KO larvae. As the efficiency of splice-blocking (SB) morpholinos can be addressed by qPCR, we desided to utilize these types of morpholinos. Alltogether, we ordered two morpholinos for *crp3* and one for both *crp2-2* and *BX548011.1*. Doses ranging from 1 to 4 ng/embryo were tested for all the morpholinos to find the maximum dose with no off-target effects affecting development and the efficiencies of the morpholinos (of optimal dose) were analyzed with qPCR. By qPCR, we saw decreased WT transcript levels at 3-4 dpi in zebrafish embryos injected with either of the two *crp3* morpholinos compared to random control morpholino injected embryos. One of the two (named as *crp3* SB in the manuscript) showed more robust decrease in *crp3* expression, and was also better tolerated (even with the highest dose) than the other and was therefore chosen for further studies. For *crp2-2* and *BX548011.1* morpholinos no effect could be demonstarted so we did not continue with them. We then performed pneumococcal infection and survival assay for *crp3* SB morholino injected and random control injected *crp2-1* KO larvae at 2 dpf and saw clear and significantly compromised survival of *crp3* SB injected larvae: RC morpholino injected *crp2*$^{tpu6/tpu6}$ larvae had 75 % survival, compared to 53 % survival of *crp3* SB morpholino injected *crp2*$^{tpu6/tpu6}$ larvae. We can therefore conclude, that *crp2-1* KO together with partial *crp3* knockdown results in hampered survival of zebrafish larvae from pneumococcal infection. This result also supports our previous suggestion of the compensating effect of *crp3* in *crp2-1* KO mutant line.

To present the new data, we have added a new figure, Fig 7 in the revised manuscript. This figure includes:

A) a graph showing the results from the dose titration test for *crp3* SB morpholino used in the survival studies

B) a graph showing the WT *crp3* transcript levels in *crp3* SB morpholino and RC morpholino injected larvae at 0-5 dpi

C) a graph showing the survival of *crp3* SB morpholino and RC morpholino injected *crp2-1* knockout (*crp2*$^{tpu6/tpu6}$) larvae from systemic pneumococcal infection.

In addition, the following modifications have been made to the text to describe the new data:

Abstract has been modified and the modified sentences on lines 37-41 now state (modifications in CAPITALS):

"Furthermore, knockout of one of the SIX zebrafish crp genes by CRISPR-Cas9 mutagenesis predisposed zebrafish larvae to a more severe pneumococcal infection, AND THE PHENOTYPE WAS FURTHER AUGMENTED BY CONCOMITANT KNOCKDOWN OF A GENE FOR ANOTHER CRP ISOFORM. THIS SUGGESTS a conserved function of C-reactive protein in anti-pneumococcal immunity in zebrafish."

Subtitle on lines 309-310 was modified to describe the new findings. It now states (modification in CAPITALS):

"Knockout of crp2-1 CAUSES A MODERATE PHENOTYPE DURING PNEUMOCOCCAL INFECTION, AND THE EFFECT IS AUGMENTED BY CONCOMITANT KNOCKDOWN OF CRP3"

The results from the morpholino studies have also been added to the *Results* section in which the new paragraph now states (on lines 382-401):

"To test whether crp3 compensates Crp2-1 deficiency, we used morpholino-oligonucleotides to knockdown crp3 expression in crp2$^{tpu6/tpu6}$ *larvae. A splice-blocking (SB) morpholino targeting the crp3 5' upstream sequence and tentatively causing the skipping of exon 1 where the start codon lies, was chosen for the study. Using a titration test, dose of 4 ng per embryo was chosen as this was the maximum dose that showed no developmental effects within the first 5 dpf in unchallenged AB larvae (Fig. 7A). To address the knockdown efficiency of crp3 SB morpholino, the amount of WT crp3 transcript in morpholino injected larvae was determined by qPCR utilizing primers which tentatively bind only to WT transcript. As a sign of knockdown, the crp3 SB morphants showed lower levels of WT crp3 transcript at 3-4 dpi, the most important time considering S. pneumoniae infection kinetics in zebrafish larvae, with residual expression of 24 % at 3 dpi (p<0.01) and 48 % at 4 dpi (p<0.05) compared to larvae injected with the same dose of random control (RC) morpholino (Fig. 7B). To test the synergistic effect of crp2-1 knockout and crp3 knockdown, crp3 SB morpholino and RC morpholino injected crp2*$^{tpu6/tpu6}$ *larvae were infected with 296 cfu of S. pneumoniae at 2 dpf and their survival was followed for three days. During the infection experiment, no signs of off-target effects affecting development could be detected in either of the groups. The survival of crp3 SB injected larvae from pneumococcal infection,*

on the other hand, was significantly lower (53 % survival) compared to the RC injected larvae (75 % survival) (p<0.05). We therefore conclude, that while the knockout of crp2-1 alone seems to have only a moderate effect on zebrafish survival from systemic pneumococcal infection, together with crp3 knockdown it results in significantly compromised survival of the larvae. These results suggest overlapping and compensating functions of zebrafish CRPs in the host response to S. pneumoniae."

A new sentence has also been added to the *Discussion* section to describe the new findings (on lines 510-513):

"Furthermore, when the crp2-1 knockout is combined with partial knockdown of crp3, the effect on survival is elevated which further supports their important role in zebrafish immune response and also indicates overlapping functions of different Crp isoforms."

A new section descriping the methods for morphant generation and quantification of knockdown efficiency has been added to the *Materials and Methods* section (on lines 604-618):

"Generation of zebrafish morphants and quantification of knockdown efficiency

A splice-blocking morpholino against the zebrafish crp3 was designed by and ordered from GeneTools LLC (Philomath, OR, USA) and its specificity for crp3 was checked with NCBI BLAST tool (https://blast.ncbi.nlm.nih.gov/Blast.cgi). The crp3 SB morpholino oligo sequence was 5'-TGCTCCAAGTCTGAAAAGGAAGAAA-3'. To determine the maximum dose of the morpholino with no off-target effects, three doses (4 ng, 2 ng and 1 ng) of the crp3 SB morpholino were microinjected into the yolk sac of 1-4 cell stage wild type AB zebrafish embryos. Prior to microinjection, morpholinos were heated at 65 °C for 10 min to ensure complete suspension. In microinjection, injection volume of 1 nl per embryo was used and the injection solution was dyed with 1 % Rhodamine dextran tracer (Invitrogen^{TM}, Thermo Fisher Scientific) to verify the success of the injection. After injection, the development and well-being of injected larvae were monitored daily for five days, with abnormal and dead embryos/larvae recorded.

To quantify the effect of crp3 SB morpholino on the levels of WT crp3 transcript, AB zebrafish embryos were microinjected as above with 4 ng of crp3 SB morpholino or random control (RC) morpholino (GeneTools LLC) and pools of 3-5 larvae were collected daily from 0 to 5 dpi for the extraction of total RNA."

To describe the RNA extraction and qPCR analysis related to the morpholino studies, two sentences in the *Materials and Methods* section have been modified and now state (added text in CAPITALS):

on lines 668-671:

"For the qPCR analysis of crp2-1, crp2-2 and crp3 expression in WT and mutant crp2^{tpu6} larvae AND OF CRP3 EXPRESSION IN CRP3 SB MORPHOLINO AND RC MORPHOLINO INJECTED LARVAE, total RNA was extracted from the pools of 3-5 larvae using the Qiagen RNeasy Plus Mini Kit with genomic DNA elimination step (Qiagen) according to the manufacturer's instructions."

and on lines 681-685:

"For the measurement of the expression levels of crp2-1, crp2-2 and crp3 in mutant and wild type crp2^{tpu6} larvae, AND THE EXPRESSION LEVELS OF CRP3 IN CRP3 SB MORPHOLINO AND RC MORPHOLINO INJECTED LARVAE, the reverse transcription of the RNA samples was done with the SensiFast™ cDNA synthesis kit (BioLine, London, UK) and the qPCR with the PowerUp™ SYBR® master mix (Applied Biosystems™, Thermo Fisher Scientific)."

In addition, the part of the section descriping the statistics methods on lines 709-711 has been modified, and now states (modification underlined):

"The differences in the gene expression levels measured by qPCR and the bacterial amounts were analyzed with Kruskal-Wallis with Dunn's multiple comparisons test in Fig 3B, Fig 5E-F and Fig 7B and with two-tailed Mann-Whitney test in Fig S1."

2) Fig S2. a) There a very strong induction of CRP genes in the AB/unchallenged (20 fold?). In particular, the expression of *crp2-2* is quite high from the mock challenge, is this a response to KCl or tricaine? There's data from showing that salt (NaCl) affect leukocyte trafficking and wound healing (see work form Philipp Niethammer and others) so this could be one explanation. Alternatively, does tricaine induce anything? b) It is surprising that Spn induces *crp2-1* instead of 2-2, and given the similarity in these genes, how confident are the authors that these reads are appropriately assigned? Or is there another explanation for this finding?

a) Figure S2, in fact, shows the normalized read counts (rather than the fold change) for zebrafish Crp-encoding genes. The read count of 20 in *crp2-2* therefore, depicts the baseline expression level of this gene. It is true, that some of the expression seen for *crp2-2*, and also other genes, in KCl-injected and tricaine-treated larvae may be explained by the treatment along with natural baseline expression in steady-state conditions, but since the same treatment (Tricaine and KCl) was done for all the experimental groups, these groups are comparable and thus we consider that interpretations of the results are valid.

b) The data analysis is done using the newest reference genome available at the time of RNA sequencing (GRCz10). According to that, the similarity between *crp2-1* and *crp2-2* cDNA sequence is 87 % indicating that they should be readily distinguished. Except for the qPCR on *crp2-1* in *crp2-1* KO larvae (in which the perfect match for primers most propably is not present), we have also been able to distinguish these genes by PCR and sequencing.

As we state in the manuscript (lines 324-326), we suggest that the different or actually opposite expression patterns of *crp2-1* and *crp2-2* (as well as *crp3)* might be due to overlapping function and compensatory effect of their gene products. This is also supported by the qPCR (and now by the new morholino experiment) results in Figure 5 and 7.

3) Line 575: Please describe methods for genotyping (primers and strategy) used to distinguish homozygous / heterozygous / wt. You note its by sanger sequencing but I'm not sure how this was

done? Were diseased larvae euthanized at some point before collection? Or checked very frequently before they decompose?

According to the reviewer's suggestion, a new section describing the genotyping methods in more detail has been added to the *Materials and Methods* section. In addition, the section describing the genotyping of infected *crp2*^{tpu6} larvae in KO mutant survival experiment has been expanded to clarify the larvae collection, handling and genotyping.

The new section added (lines 589-603):

"Genotyping of CRISPR mutants

*At all stages of the mutant line production, genotyping of the adult fish was carried out from the tailfin DNA by Sanger sequencing. Specifically, fish were anesthetized with 0.02 % Tricaine, and a small piece of tail fin was cut off with a scalpel blade. Tail fin tissue was lyzed for 4-24 h at 55 °C in lysis buffer (10mM Tris pH 8,2, 10mM EDTA, 200mM NaCl, 0.5% SDS, 200µg/ml Proteinase K). DNA was precipitated for 1h-24h at -20˚C using two volumes of ethanol after which DNA was pelleted by centrifuging at 16,000g for 10min. The pellet was washed with 70 % ethanol and resuspended in water. The genomic site containing the mutagenesis target site was amplified using DreamTaq™ Hot Start DNA Polymerase (Thermo Scientific™) and sequenced using the BigDye™ Terminator v3.1 Sanger sequencing Kit (Applied Biosystems™, Life technologies, Carslbad, USA). Primers used in the genotyping are listed in **Table 7.** To distinguish homozygous and wild type fish, the obtained sequence was compared to the wild type sequence using Clustal Omega (147,148). To analyze the mutation site in the heterozygote fish, CRISP-ID v1.1 web application (162) for detecting CRISPR induced indels was used. Alternatively, the two overlapping sequences (one from the WT allele and the other from the mutant allele) were manually separated from the sequencing chromatogram."*

The expanded description on lines 645-650 now states (modifications in CAPITALS):

"In the experiment, the survival of the infected larvae was followed for 72 hours, during which THE LARVAE WERE CHECKED TWICE A DAY. TERMINALLY DISEASED AND DEAD LARVAE WERE COLLECTED, EUTHANIZED IF NECESSARY, AND FROZEN FOR LATER GENOTYPING. FOR THE GENOTYPING, THE GENOMIC DNA EXTRACTION FROM WHOLE LARVAE, TARGET SITE AMPLIFICATION, AND SEQUENCING, WERE DONE AS DESCRIBED FOR ADULT FISH GENOTYPING IN THE SECTION GENOTYPING OF CRISPR MUTANTS."

4) Line 644: Mann Whitney tests are only appropriate for comparison of two experimental groups. Many of the analyses here have more than two groups being compared and a Mann Whitney should not be used: Fig 3, 5. (Kruskal-Wallis, ANOVA, or other tests may be appropriate depending on the comparisons and normality of the samples). No stats are included in Fig 2 or 4, but should be.

According to reviewer's suggestion, the statistics in Figures 3 and 5 were redone with Kruskal-Wallis test with Dunn's multiple comparisons test. In Figure 5, the idea of the results was to show the induction of genes in WT and mutants upon infection and, therefore, comparisons were only done to the 0 h time point (representing the state before infection) of the same gene. The figures were modified accordingly and the legends updated on lines 1199 and 1227.

None of the differential expressions in RNA sequencing were statistically significant when analysed with DESeq2 (Wald test + correction for multiple testing). This is why the differential expressions were verified with qPCR for the most relevant genes (8 replicates, Figure S1). To retain the consistency in the manuscript, insignificance is not shown in the figure. However, for clarity, the following sentences were modified/added:

A modiefied sentence in *Statistical Analyses* section on lines 711-713 now states (added text in CAPITALS:

"In each analysis, a p-value of <0.05 was considered as statistically significant AND ONLY P-VALUES <0.05 ARE SHOWN IN THE FIGURES."

The following sentence has been added to the legends of Figure 2 (lines 1187), Figure 4 (lines 1207-1208) and Figure S2:

"The differential expressions did not reach statistical significance"

5) The genetic screen used a cross with TL, is it possible differences in this and the AB background lead to your differences / lack of recapitulation in the CRISPR KO of *Crp2-1*? Why were screens done in TL and targeted deletion in AB? Unless I am misunderstanding the methods here, this is a major concern if the RNAseq is done in TL background but compared to AB?

The screen lines were done in AB background and outcrossed to TL to get F1 and F2 generation fish as described in *Materials and Methods* section (lines 566-571). The reviewer is right by saying, that AB line is not a perfect negative control for survival nor RNAseq, but instead GFP-negative siblings from each line would have been. Since those siblings were rarely available (either all larvae in the patch were GFP-positive or GFP-negative siblings could not be distinguished from moderately GFP-positive siblings due to autofluorescence) we chose to use AB as a control as this was the original background.

In addition, all the screen lines had the same "ABxTL" background but only few lines showed susceptibility to pneumococcus. Importantly, in the survival assays, the lines with comparable survival to wild types acted as additional negative controls and helped to distinguish the most susceptible lines. To emphasize the fact that the screen lines with survival comparable to AB acted as additional controls, the following sentences have been modified:

on lines 257-262:

"In the survival assays, 2 dpf zebrafish larvae from each mutant line were infected with S. pneumoniae and their survival was compared to the AB wild type line and the other mutant lines in the same experiment. A total of 58 survival experiments were conducted (bacterial doses ranging from 30-570 cfu between experiments). In each experiment, a wild type control and at least three mutant lines were included to distinguish hypersusceptible lines."

and on lines 262-265 (modifications in CAPITALS):

*”IN THE INITIAL SCREENING, ABOUT 10 % OF THE LINES APPEARED TO HAVE ALTERED SUSCEPTIBILITY TO PNEUMOCOCCAL INFECTION, AND one of the lines in particular, named as mutant94, showed a notably decreased survival of 28 % after S. pneumoniae challenge, compared to the final survival of 74 % (p<0.0001) in challenged wild type (AB) controls (**Fig 3A**).”*

The fact that most of the screen lines having the ABxTL background had normal survival to *S. pneumoniae* also proves that TL background (which is missing from the *crp2-1* KO line) cannot explain the more moderate phenotype of *crp2-1* KO line.

All in all, we consider that appropriate controls (related to genetic background) are used in different experiments: in mutant94 experiments a separate wild type AB line was used while WT siblings with identical genetic background (including possible off-target mutations) were used in *crp2-1* KO survival. We also think, that due to different negative controls used, as well as different genetic backgrounds of mutant94 and *crp2-1* KO themselves, these two survival experiments cannot be compared directly.

Minor concerns:

6) Line 72: “one of the most important pathogens in humans” is over stated. Please modify and/or provide a citation for why this is considered to be the case.

To avoid overstating, the sentence on lines 72-73 has been modified and now states (modifications in CAPITALS):

”Streptococcus pneumoniae (pneumococcus) is a Gram-positive opportunistic bacterium AND AN IMPORTANT HUMAN PATHOGEN.”

7) Line 76: “respiratory tract infections, such as acute otitis media” – otitis media is not a respiratory tract infection

The sentence on lines 75-77 has been modified and now states (modification in CAPITALS):

”It is one of the leading causes of community-acquired pneumonia but also a common cause of milder upper respiratory tract infections, AND acute otitis media (1,2).”

8) Line 77-78: It sounds like you are suggesting that otitis media and sinusitis can lead to bacteremia and meningitis? Typically this proceeds from a pneumonia. Please clarify and provide citations either way.

To avoid such intepretation, the sentence on lines 77-78 has been modified and citation added (modification in CAPITALS):

"Occasionally, PNEUMONIA may manifest as an invasive disease, and lead to the outbreak of life-threatening bacteremia and meningitis (1,2)."

9) Line 79-80: "Moreover, pneumococcus is responsible for about a half a million child deaths each year making it one of the most common causes of lethal infection at young age" – This statement again seems a bit over stated (and I don't think its necessary for the authors to over emphasize). The papers cited do not state that claim, and one estimates between 200-300k per year, with a note that it could be as high as 500k (in 2015, and there has been increasing PCV vaccination as of 2020). Other estimates from the WHO seem closer to 300k, so rounding to "half a million" and "the most common cause" is a bit strongly worded.

We agree with the comment, so to avoid overstating, the first chapter in the *Introduction* section has been modified and the citations have been updated. The modified section on lines 78-84 now states (modification in CAPITALS):

"Due to the high incidence of pneumococcal infections, this pathogen is a major cause of morbidity especially in children and the elderly, AND ACCOUNTED FOR OVER A MILLION DEATHS GLOBALLY IN 2016 (3). WHILE THE GLOBAL USE OF PNEUMOCOCCAL CONJUGATE VACCINES HAS REDUCED THE COLONIZATION, PNEUMONIA, ACUTE OTITIS MEDIA, AND INVASIVE DISEASE CAUSED BY THE VACCINE SEROTYPES, THE INCREASED PREVALENCE OF NON-VACCINE SEROTYPES AND THE HIGH INCIDENCE OF ANTIBIOTIC RESISTANT ISOLATES OF S. PNEUMONIAE RETAIN THIS PATHOGEN AS A MAJOR HEALTH BURDEN WORLDWIDE (4–6)."

10) Line 97-99: This paper also demonstrates a link to complement deficiencies which is relavent to your conclusions. It also states theres not a big link with defects in phagocytosis though (which you go on to emphasize in line 102).

This citation (Picard et al., 2003) indeed demonstrates a link between complement deficiencies and pneumococcal infection and, therefore, the citation is added to the site where the importance of complement deficiencies is mentioned, on line 106. In addition, "C3" is added in the example list on line 106, as C3 deficiencies, in particular, are highlighted in Picard et al., 2003.

11) Line 105: NOD should be capitalized (not Nod) as its an abbreviation

According to reviewer's suggestion, "NOD" has been capitalized on line 104.

12) Line 109: Citation 25 concludes there is no association with MBL2. Please double check citations to ensure conclusions are properly reported.

The citation was double checked. In the citation Kloek et al., 2019, the authors do infact conclude that the MBL2 variant alleles B, C and D were significantly associated with susceptibility to pneumococcal

infection, but not with the outcome of pneumococcal infection. Therefore we decided to keep the sentence on lines 106-107 which has been modified and now states:

"A recent meta-analysis also confirmed two susceptibility polymorphisms, one in MBL2 and one in CD14 (21)".

13) Line 110: "As a result, the understanding of the genetic basis of pneumococcal disease remains limited." The authors suggest this is due to poorly conducted meta analyses (this is what is concluded in citation 25, which the authors build from). An alternative conclusion is that the polymorphisms leading to susceptibility are variable, as might be predicted by the variability in disease outcome, severity, etc seen in clinical infection (humans and is recapitulated in animal models). The authors seem to be suggesting that zebrafish or animal models in general might be a better way to assess this question, but ultimately, the only way to determine what human genes are important is to look in humans. I think the author's foundation /intro would be much stronger by emphasizing the power of zebrafish and showing that it does in fact correlate with many findings in humans, and is therefore a useful tool, instead of being over critical of human data.

We agree that this site of the text is unclear and such a message was unintended. Therefore, the two sentences on lines 106-110 were modified and now state:

"A recent meta-analysis also confirmed two susceptibility polymorphisms, one in MBL2 and one in CD14 (21). However, in general, individual association studies have often given conflicting results emphasizing the complexity of the genetic basis of pneumococcal disease and, therefore, further studies on the factors affecting disease susceptibility and severity are needed."

In addition, we hope that together with the above mentioned modification, the following sentence on lines 111-113 would prevent the unwanted intepration that animal models should be preferred over human studies as we do not wish to give such message:

"Along with the studies on genetic variation in humans, transcriptome analyses and genetic screens conducted in animal models are useful in providing insights into the genetic basis of host response to infection at a genome-wide level."

14) Line 137: "As a result, we found that the innate immune response in pneumococcal infection in zebrafish is well conserved which further validates the use of this model in the evaluation of the host-pathogen interactions during pneumococcal infection". Well conserved with what? Presumably humans? The authors do not say what they are comparing to or what parts are conserved.

This perhaps too generic phrase on line 136-139 has been modified to describe the level of similarity in zebrafish and mammals, and now states (modifications in CAPITALS):

"As a result, we found that SIMILAR MECHANISMS OF INNATE IMMUNITY, SUCH AS COMPLEMENT-MEDIATED CLEARANCE, ARE IMPORTANT IN PNEUMOCOCCAL INFECTION IN ZEBRAFISH AS IN MAMMALS, which further validates the use of this model in the evaluation of the host-pathogen interactions during pneumococcal infection."

15) Line 152: "time point of 18 hpi represents the acute stage of the pneumococcal infection in zebrafish larvae" – Can you clarify what you mean by "the acute stage"?

As suggested by the reviewer, the term "acute stage" has been replaced with a more informative description and the new sentence on lines 152-155 now states (modifications in CAPITALS):

"According to our previous studies IN ZEBRAFISH LARVAE (28), a time point of 18 hpi represents A STAGE OF PNEUMOCOCCAL INFECTION AT WHICH THE BACTERIA ARE REPLICATING RAPIDLY, THE HOST INNATE IMMUNE RESPONSES HAVE BEEN ACTIVATED AND THE SIGNS OF INFECTION (E.G. LACK OF MOVEMENT) APPEAR, and was therefore chosen for the analysis."

16) Line 162-164: What percentage of the genome is this? How does this compare to the number of genes altered in mouse or human acute infection?

The given number represents the number of up- and downregulated genes which meet the chosen criteria (fold change ≥3 and mean normalized read count of ≥20) and not the total number of differentially expressed genes. While it would, indeed, be interesting to compare the number of differentially expressed genes to those from other models, the numbers (or percentages) themselves are not comparable as in every study different criteria (for example fold change, minimum read count, p values and variation) can be set. Here, the goal was to highlight the most important mechanisms in host response and therefore the cutoff was set relatively high.

17) Line 185: Table 1: What do the commas signify in the fold change? Were there replicates that were 28-fold and 9-fold (for the first line, c3a.2 as an example). Or do the authors mean 28.9-fold (in which case this should be a decimal).

The numbers in the fold change column signify decimal, and therefore, the commas in Table 1 have now been replaced with decimal points.

18) Table 1 and experimental design: the legend states fold change vs uninfected. Were these truly uninfected or mock-infected (PBS control, still received tricaine and a mock injection?)

We have used mock-infected controls. We have checked that the treatments done to unchallenged control larvae are described and can be found in *Materials and Methods* section. The description on lines 640-642 states:

"The unchallenged control groups were similarly treated with either 0.2 M KCl (infection experiments related to the genetic screen and mutant94) or PBS (infection experiments related to crp2^{tpu6})."

19) Line 193: You state the response is well conserved. Please describe how. (what are you comparing to, a septic infection in mouse and blood transcriptome at a similar timepoint? Or peripheral response in humans with invasive infection during hospitalization?) Please include citations for the comparison to support the claim. Complement function is based on protein mostly, not sure how the transcriptional response compares (as is shown here).

We agree that the phrase "response is well conserved" is rather generic and lacks the details of the conditions and models beeing compared.

To avoid the confusion with the term "conserved", we have modified the particular sentence on lines 195-196 which now states (modifications in CAPITALS):

"Our transcriptome data PROVIDE EVIDENCE FOR THE ACTIVATION OF AN innate immune response to pneumococcal infection at the level of gene expression."

In addition, the subtitle on lines 193-194 has been modified accordingly, and now states (modifications in CAPITALS):

"The transcriptome analysis INDICATES AN innate immune response to pneumococcus in zebrafish, WITH CONSERVED PATHWAYS"

20) Line 204-206: Two homologs of CRP in zebrafish associated with the activation of complement in humans were induced? This should be two sentences, one saying there are 2 zebrafish homologs of CRP that are induced, and another one explaining the function of CRP in human.

As suggested by the reviewer, the sentence has been divided in two, and the new sentences on lines 206-209 now state:

"In addition, two zebrafish homologs for the human gene encoding C-reactive protein (crp2 and crp3, ENSDARG00000056498 and ENSDARG00000042613, respectively) were induced upon infection in zebrafish larvae. CRP IN MAMMALS IS KNOWN TO RECOGNIZE S. PNEUMONIAE AND TO HAVE MULTIPLE FUNCTIONS IN ANTIPNEUMOCOCCAL RESPONSE INCLUDING the activation of the complement system and phagocytosis (53)."

21) Line 208-211: The authors could do more to explain the function of these genes (RNAse and chitinases are not obviously antimicrobial) – this is not obvious how they might function, and since the article is seeking to characterize the innate immune response to Spn, this is an important point.

The reviewer is right by saying that the description of gene function in this chapter is limited, but since the functional conservation of many of the genes listed there is not yet known, it is rather speculative

to discuss their role in *S. pneumoniae* infection in zebrafish in detail. Instead, we have kept the focus in what is known about these genes in zebrafish, and if no zebrafish studies are available, we have summarized their relevant function in mammals. However, to avoid oversimplifying the gene function and to add the lacking information (the other known functions of zebrafish RNAsels), the following sentence on lines 212-214 was modified as follows (modification in CAPITALS):

"These included the genes for antimicrobial peptide hepcidin (hamp) (54) and FOR ONE OF THE RIBONUCLEASE A SUPERFAMILY MEMBERS (ribonuclease-like 3, rnasel3) WHICH HAVE BEEN SHOWN TO HAVE ANTIBACTERIAL, ANGIOGENIC AND NEUROTROPHIC ACTIVITY IN ZEBRAFISH (55,56)."

We have checked that, related to chitinases, also the other known functions than antimicrobial activity are mentioned (lines 214-216).

22) Line 248: "gene-breaking" may not be widely recognized and would be helpful to define this genetic approach.

We agree that "Gene-breaking" may not, indeed, be widely recognized but instead a specific name of this particular method. As the "gene-breaking" part of the method (production of truncated protein fused with mRFP upon integration within a gene) was not utilized in this study (mutants regardless of the insertion site were selected based on GFP expression), we think it would be more confusing than informative to define the "gene-breaking" part here in detail. We, therefore, removed the term entirely from the sentence, and added a new sentence to clarify the selection of mutants.

The modified sentence on lines 251-253 now states:

"To identify novel genes affecting the innate immune response to pneumococcal infection, we carried out A FORWARD GENETIC SCREEN IN ZEBRAFISH UTILIZING A TRANSPOSON-BASED MUTAGENESIS METHOD CREATED BY CLARK ET AL. (2011) (75)."

And the added sentence on lines 256-257:

"Zebrafish larvae showing GFP expression as a sign of the presence of one or more randomly located mutations in their genome, were selected for the assays."

23) Line 252: Inocula is 30-570 CFU but in the legend it says ~300 CFU. Are you only showing the results from the intermediate dose or are these results in figure 3 polled from multiple experiments? If multiple, what was the range of CFU? Particularly for panel B, where we are comparing CFU in larvae, this varies drastically based on inoculum and is important to note.

Results shown in the original Figures 3A and 3B consis of multiple experiments. Therefore, the average bacterial dose ~300 cfu calculated from all the screen survival experiments has been replaced with a

range of cfu *"35-440 cfu"* used in the experiments included in the figure A (line 1194). Of note, these images are representative and when the lines were screened for altered survival they were always compared to the wild type line and other mutant lines within the same experiment to exclude the effect of variation in bacterial dose as is now described in more detail on lines 635-637:

"To avoid the influence of varying doses, all groups within a single experiment were infected with the same bacterial solution and only groups from the same assay were compared to find differences in survival or bacterial count."

As it is true, that the variation in inoculum has a great effect on bacterial counts measured in Figure 3B, we have replaced the figure with another containing results from only one experiment with a dose of 505 cfu/larva (n=8). The Figure 3 legend was also modified accordingly (lines 1196, 1197).

Of note, for consistency and clarity, we have replaced the dose range on lines 349 and 371 to represent the average dose (obtained from parallel injection samples) in single experiment. Similar modifications are done in the legends of Figures 5 and 6 (lines 1226 and 1232).

24) Figure 3B. It would be nice to change the dots to outline (not filled) so you can see how many samples there are. Currently its hard to distinguish overlapping dots, particularly in AB and mutant14

The dots in Figure 3B have been replaced with circles according to reviewer's suggestion and the figure legend has been modified accordingly (line 1197).

25) Line 363: Are these mutants more resistant than other lines, (dose increased from 30-570 to 1412-1880). This is anywhere between a 3-fold to 63-fold increase in dose, either is quite significant in most models.

The dose range is indeed relatively high in the survival experiments and the reported dose (1412-1880) in *crp2-1* KO survival study particularly high.

Due to the variation in bacterial dose as well as other factors between the experiments (e.g. the genetic backgrounds of the two types of mutant lines) groups from different experiments cannot be compared and thus we unfortunately cannot comment on whether KO line is more or less resistant than the mutant lines in the screen.

As the variation between experiments cannot be completely avoided, it needs to be taken into account in the experimental design. To describe the possible sources of variation in bacterial dose between experiments and to highlight how the variation was taken into account, we have included the following sentences to the text:

on lines 631-637:

"Due to variation in bacterial growth rate, accuracy of absorbance measurement, slightly varying volumes of bacterial solution released by the injector, accuracy of quantitative plating etc., relatively big variation in bacterial doses between experiments (30-570 cfu in infection experiments related to the genetic screen and mutant94 and 296-1,646 cfu in infection experiments related to crp2^{tpu6}) was seen. To avoid the influence of varying doses, all groups within a single experiment were infected with the same bacterial solution and only groups from the same assay were compared to find differences in survival or bacterial count."

26) Larvaculture – please describe how the zebrafish were raised from 0-6dpf (presumably in an incubator with constant darkness?). Please note if light dark cycling was used during early development or during infection studies.

The details requested by the reviewer have been added to *Materials and Methods* section. The modified sentence on lines 535-539 now states (added details in CAPITALS):

"Briefly, zebrafish embryos and larvae were kept in E3-medium (5 mM NaCl, 0.17 mM KCl, 0.33 mM $CaCl_2$, 0.33 mM $MgSO_4$, 0.0003 g/l Methylene Blue) IN AN INCUBATOR at 28 °C, WITH A LIGHT/DARK CYCLE OF 14/10 HOURS until 6 dpf after which they were moved to a filtered flow-through system with the same light/dark cycle and fed once a day with GEMMA Micro 75-500 (Skretting, Stavanger, Norway) or twice a day with SDS 100-400 (Special Diets Services, Essex, UK)."

In addition, to describe the conditions during the experiments in more detail, the following sentence has been added (line 542):

"During the experiments, embryos and larvae were maintained in corresponding conditions."

27) Line 563: What is sequence type 205?

The sequence type refers to highly invasive clonal type (number 205), but since TIGR4 is widely used and genome sequenced reference line of *S. pneumoniae*, such a detail may not provide any additional information. To avoid confusion, the "*sequence type 205*" was removed from line 620.

28) Line 565: please describe brief culture media and methods for Spn

According to the reviewer's suggestion, the paragraph (on lines 622-637) in *Materials and Methods* section is expanded to describe the methods for *S. pneumoniae* culture, harvest and the preparation of injection solutions. The new part of the paragraph now states:

"Briefly, bacteria were grown overnight on 5 % lamb blood agar plates (Tammer BioLab Ltd, Tampere, Finland) at 37°C and 5 % CO_2 and after that, in liquid culture in Todd Hewitt broth (Becton, Dickinson and Company, New Jersey, USA) supplemented with 0.5 % yeast extract (Becton, Dickinson and Company) until mid-logarithmic growth phase determined by optical density at a wavelength of 620 nm. Bacterial cells were harvested by centrifuging for 10 min at 4000 rpm (rounds per minute). Using

the theoretical bacterial density in the liquid culture (10^8 cells/ml), the bacterial pellet was suspended in desired volumes of 0.2 M KCl (infection experiments related to the genetic screen and mutant94) or PBS (infection experiments related to crp2^{tpu6}) to obtain the desired bacterial dose. The actual number of bacteria in the injection solution was verified by plating samples of single injections on 5 % lamb blood agar plates (Tammer BioLab Ltd). Due to variation in bacterial growth rate, accuracy of absorbance measurement, slightly varying volumes of bacterial solution released by the injector, accuracy of quantitative plating etc., relatively big variation in bacterial doses between experiments (30-570 cfu in infection experiments related to the genetic screen and mutant94 and 296-1880 cfu in infection experiments related to crp2^{tpu6}) was seen. To avoid the influence of varying doses, all groups within a single experiment were infected with the same bacterial solution and only groups from the same assay were compared to find differences in survival or bacterial count."

29) Line 566: Why were bacteria resuspended in KCl for some experiments and PBS in others

At the time when this project started, KCl and PBS were both widely used in various infection studies. In our studies, KCl was originally used in *S. pneumoniae* solutions but during the project it was replaced by more physiological salt solution, PBS. According to our tests, change of the solution did not affect the bacteria nor the fish. We have checked that the *Materials and Methods* section includes the following clarifications whether KCl or PBS was used in a particular experiment:

on lines 627-629:

"…the bacterial pellet was suspended in desired volumes of 0.2 M KCl (infection experiments related to the genetic screen and mutant94) or PBS (infection experiments related to crp2^{tpu6}) to obtain the desired bacterial dose."

and on lines 640-642:

"The unchallenged control groups were similarly treated with either 0.2 M KCl (infection experiments related to the genetic screen and mutant94) or PBS (infection experiments related to crp2^{tpu6})."

30) Line 568: What agar base was used (Columbia agar+ lamb blood, or TSA agar+ lamb blood etc?)

The agar plates were obtained from a company (Tammer Biolab Ltd) and such details are unfortunately not public.

31) Line 572: A matter of preference but I would call these mock challenged or uninfected (since you did in fact do control injections).

We appreciate the reviewer's comment and agree, that there are several good options to name the control group. To avoid confusion, we have checked that the treatments done to the control group are appropriately described in *Materials and Methods* section (lines 640-642).

32) Table S1, S2, S3, S4, S5, S6 similar comment as table 1. I believe the authors should have a decimal point instead of a comma in the fold-change column.

As in Table 1, the numbers in the fold change column signify decimal, and therefore, the commas in Tables S1, S2, S3, S4, S5, and S6 have been replaced with decimal points.

33) Fig S1. Please expand the lower axis and add more tickmarks. Somewhere between 0 and 50 fold change is a huge range!

According to the reviewer's suggestions, the length of the bottom Y-axis has been increased from 80 % of the whole axis to 90 %. In addition, the length of the whole axis is increased to almost double the original axis and more tickmarks are added.

34) In case the authors are not familiar, this pre-print may be in interest:
https://www.biorxiv.org/content/10.1101/2022.02.10.479878v1

Very interesting indeed. As it is, in fact, very relevent to the topic of our manuscript, citation to this pre-print has been added on line 428.

Comments from Reviewer 2

The paper by Saralahti et al. describes how the innate immune system of zebrafish larvae is affected by Streptococcus pneumoniae infection and highlights how the mechanisms involved are conserved between zebrafish and human. To this end, they used an established infection model of zebrafish embryos and performed a whole-genome transcriptomic analysis to identify the altered pathways following pneumococcal infection. They found protein coding genes as well as non-coding RNAs presenting a differential gene expression level, some were upregulated, and others were downregulated. Most of these genes were associated with the immune response, but some were involved in metabolic pathways or had unknown functions.
Most importantly, a comparison to the human innate immunity-related genes expressed during pneumococcal infection showed well conserved mechanisms, notably components of the complement and in particular the complement activating protein CRP - for which two homologs exist in the zebrafish (crp2, *crp3*), had their expression altered.
Next, the authors further validated their observations through a forward genetic approach where they screened 126 generated zebrafish mutant lines for susceptibility to pneumococcal infection: they ended up with one mutant (mutant94) that showed exceptionally low resistance to infection compared to AB control and other mutant lines. Data from RNA-sequencing performed on this mutant revealed an important impairment of crp2_1 gene expression (a human CRP homolog). To further confirm the previous results and investigate the mechanisms in more detail, the authors decided to generate a crp2_1 knockout zebrafish line using CRISPR-Cas9, resulting in a truncated *Crp2-1* protein, and proceeded with the same pneumococcal infection protocol. Here again, they observed a poorer survival rate of the knockout mutant line compared to AB control (although less dramatic than what they observed in mutant94), which confirmed the importance of the C-Reactive Protein in immunity against pneumococcal infection.
Although the importance of the innate immune system following infection in zebrafish has been

highlighted in previous studies (Saralahti et al. 2014), the high-throughput approach used here brought a global view that also allowed the discovery of new potential effectors previously unknown to be involved in the response to pneumococcal infection in zebrafish larvae. Because evidence of the conservation of the mechanisms related to innate immunity between zebrafish and human was shown, the results also suggest a possible conservation of the other pathways, possibly in humans, which emphasizes the high relevance of the use of the zebrafish model for this type of studies.
The authors introduced the public health issue very nicely making the relevance of the model and the study very convincing. The story was described in a clear manner, easy to follow and understand, with simple but robust and very well executed approaches as well as the use of a vast array of relevant techniques. I think the authors proved their point with a nicely designed study.

Minor concerns

1) Line 213 to 215: I first thought the phrase was unfinished and a verb was missing at the end but then realized the misuse of “comprised of”, which is the case also on line 241 and can make these sentences difficult to understand.
I think “be” should be added to make it into the verbal expression “to be comprised of xyz” or “of” needs to be removed if the verb “to comprise xyz” is used instead.

The two sentences have been modified to replace the ”comprised of” with correct and more suitable terms. The modified sentences now state (modification in CAPITALS):

on lines 219-221:

”The category of the immune signaling and regulation, on the other hand, INCLUDED the lipid transport gene apoa4b.3, the G-protein coupled receptor gene gpr84 (66) and a member of lymphocyte antigen 6 family (ly6m2).”

and on lines 246-248:

*”In addition, the ANALYSIS also REVEALED multiple UPREGULATED protein coding genes and non-coding RNAs (**Table S1 and S3**) with unknown function, also serving as candidates for further functional characterization. ”*

2) The 2 paragraphs from line 207 to 233: I think one should refer to Table 1 as the last figure mentioned was Fig2, so it can get confusing since everything discussed is about Table1.

Citations to Table 1 were indeed missing in these two paragraphs. To have an appropriate spot for the new citations, the two sentences on lines 210-214 have been modified as follows (modified part in CAPITALS):

*”Besides the induction of Crp-encoding genes and complement genes, the early response to pneumococcus in zebrafish was characterized by the expression of OTHER GENES PREVIOUSLY ASSOCIATED WITH INNATE IMMUNE RESPONSE TO PATHOGENS (**TABLE 1**). THESE INCLUDED the*

genes for antimicrobial peptide hepcidin (hamp) (54) and for one of the Ribonuclease A superfamily members (ribonuclease-like 3, rnasel3) which have been shown to have antibacterial, angiogenic and neurotrophic activity in zebrafish (55,56)."

In addition, citations to Table 1 were added to lines 212 and 226.

3) Another general comment I have is regarding the placement of the citations: in some cases (for example line 223 to 233), the citations are left at the end of the sentence even though it ends with a description of the authors' own results from what I understood. I believe the citation in such examples should be moved higher up, right after the cited part, to avoid confusions.

We have checked the sites of citations throughout the text and moved three sitations higher up in the sentence according to reviewer's suggestions. The citations moved are now on lines 223, 231, and 235.

4) Lines 248-250: very impressive massive work!

We agree with the reviewer, it was a massive amount of work indeed.

5) Lines 252 – 253 : 30-570 cfu, Why such a big range of CFU? Were different CFUs tested or was it just meant to be 300-570?

The true range of cfus was the given 30-570 cfu. While this is, indeed, quite a big range, we feel that observed variation in the bacterial dose between infection experiments (all together 58 experiments in the screen) is understandable and results from the variation in bacterial growth and the accuracy of spectrophotometer and microinjector as well as inaccuracies in the quantification method itself etc (now described in the revised manuscript on lines 631-637).

Importantly, within this range, the variation in the dose did not hamper the distinguishing of the hypersusceptible lines from the lines with WT phenotype. It is important to note, however, that due to the variation in bacterial dose (but also other factors) between experiments, results obtained from different experiments cannot be directly compared. In addition, in a single experiment, all the groups received the same bacterial dose.

Please see the response to comment 25 of reviewer 1 for the changes made to the manuscript to better emphasize the variation in bacterial counts and how this was taken into account in experimental desing.

6) 261 to 267: The resistance vs tolerance experiment. I get the point and it's very well explained, but I'm wondering, even though it is quite clear that mutant94 has impaired resistance, couldn't it

also be that in addition to that it also has impaired tolerance? (because they also die more than the other hypersusceptible lines according to Fig 3A) I don't think this requires any further experimental proof, but just a point maybe to add in the interpretation of the data?

This is a good point and to avoid oversimplification, the particular paragraph on lines 269-275 is modified as follows (modifications in CAPITALS):

*"To determine whether the hypersusceptible mutants had defects in resistance or in tolerance, we measured the bacterial burden in the infected larvae at 18 hpi. As shown in **Fig 3B**, the mutant94 larvae had elevated bacterial burden compared to AB larvae (p<0.001) AND THUS SHOWED DECREASED ABILITY TO CONTROL BACTERIAL GROWTH. In contrast, bacterial burden in the other hypersusceptible lines did not differ from the bacterial burden in the wild type larvae. These results indicate that the POOR SURVIVAL OF MUTANT94 LARVAE AFTER PNEUMOCOCCAL CHALLENGE IS MAINLY DUE TO compromised resistance against pneumococcal infection while the mutant450 and mutant14 larvae RATHER have poorer tolerance."*

7) Line 363: why more than 1000 cfu are used?
I am also curious as to why different cfu were used throughout the study? (500 cfu in Fig 2, 300 cfu in Fig3 and 1600 cfu in fig6). I don't recall this point being addressed, but my apologies if I missed it.

Please see the response to comment 25 from reviewer 1 and to comment 5 from reviewer 2.

8) Line 370 - Although an in-depth characterization of the truncated protein in the KO mutant might be complicated and probably unnecessary here, maybe on the other hand a further genome analysis of the mutant94, sequencing, genotyping, might be of interest to actually identify the other components potentially involved together with crp2_1 and impaired in this mutant? But I understand these approaches can be complicated and time-consuming, when in my opinion the authors proved their point already in this paper, but it could be an interesting follow-up for further studies maybe.

We thank for this valuable comment. A whole genome analysis of mutant94 would be great in showing in detail the genetic changes in this line and indeed would be an interesting follow-up for this study. However, whether the genetic changes affect the expression of important genes, can only be revealed by transcriptome analysis and that is why this approach was selected to find factors causing mutant94 susceptibility to pneumococcal infection. Along with *crp2-1*, the transcriptome analysis did reveal other possible factors, but we consider that the detailed study of those was out of the scope of this study.

9) Fig 4: very clear and to the point results, but just curious, why was the unchallenged mutant not included?

The unchallenged control would have been a great add to the analysis, but since we were interested in the differences between challenged WT and challenged mutant, it was not included.

10) Very nicely discussed topic, with a clear take-home message, I like the unexpected opening to a whole new field (evolution of vertebrate immune system)!

We want to thank the reviewer for this compliment and also the accurate suggestions to further improve our manuscript.

Additional changes made to the manuscript

1) A new author (Maiju Junno) has been added to the list of authors as she was instrumental in carrying out the new experiments (line 5)

2) Organization of Figure 5 has been modified to meet the PLOS Genetics reguirements for Figure dimensions

3) A sentence in Figure S1 legend was modified to correct a mistake and it now states (modification in CAPITALS)

”A DOT represents relative gene expression in A single POOL OF wild type larvae (AB/challenged) and mutant94 larvae (mutant94/challenged) infected with ~500 cfu of S. pneumoniae compared to the AB larvae injected with KCl.”

4) Reference list on lines 725-1161 was modified

5) Minor textual modifications were done due to language check or PlosGenetics technical check. These changes have been marked with ”track changes” option in the ”Revised Manuscript with Changes Highlighted” file.

Sincerely,

Mika Rämet, MD, Ph.D.
Professor of Experimental Immunology
Faculty of Medicine and Health Technology
Tampere University
Finland
mika.ramet@tuni.fi
Tel. +358 50 433 6276
