# Supplementary material for: Characterization of the innate immune response to Streptococcus pneumoniae infection in zebrafish
Source: PLoS Genet. 2023 Jan 9;19(1):e1010586. doi: 10.1371/journal.pgen.1010586 (PMC9858863; doi:10.1371/journal.pgen.1010586)
Supplement: Attachment — Submitted filename: Spn reviewer comments.docx [file pgen.1010586.s011.docx]

This study leverages a zebrafish larval model of Streptococcus pnuemoniae infection to identify genes involved in innate immune response to sepsis. The authors use transcriptomics to characterize the acute inflammatory response to Spn in larvae, demonstrating that immune pathways activated in zebrafish are similar to the immune pathways that are well known to be activated in human and mammalian infections – such as complement factors. The authors then perform a forward genetic screen to identify mutants with increased susceptibility to Spn infection and identify a hit in the gene encoding C-reactive protein (CRP). CRP is well known clinically as an important sign of acute inflammation, therefore demonstrating consistency and conservation of the response between zebrafish, a cold-blooded vertebrate and humans. The experiments are well designed and provides an extremely useful foundation to validate this underutilized model. However, ultimately, the putative role for CRP in defense against Spn was not genetically validated, as the targeted CRISPR mutant did not recapitulate the severe phenotype of the mutant identified in their screen. Thus as it stands, this work has the potential to be useful to the field, but the conclusions of the role of Crp2-1 in zebrafish is not sufficiently supported by experimental evidence – this is a major concern that should be addressed before publication. At a minimum, the authors should provide genetic evidence of the factor responsible for susceptibility in their line (perhaps crp3 or crp2-2, or a second gene). Generating double knockout lines would be the most compelling but is a significant amount or work / time to complete. Instead, the authors may be able to address these concerns with morpholino knockdowns. Additionally, this paper would be improved with experimental mechanisms or validation beyond transcription (i.e. protein level or functional killing against S. pneumoniae) though this is not a critical for publication in my opinion. Specific comments are included below.

**Major concern:** It is disappointing that after all the work making the various lines, the phenotype is not recapitulated in the CRISPR deletion of crp2-1. Based on the challenges with qPCR, the possible compensatory effect of the other crp’s as the authors point out, or the possibility that the mutant94 had another mutation, this is a critical concern that should be addressed. As it stands, the title of this section (line 302: Knockout of crp2-1 renders zebrafish larvae more susceptible to systemic pneumococcal infection) is not supported by their data. Although there is technically a very small but significant difference in survival, this looks to be something that’s only possible with very high n and only when analyzed at the late time point. It certainly does not recapitulate the phenotype seen in the mutant94.

In lieu of making a double knockout line, the authors might try using morpholinos to knock down crp3 in the crp2-1^tpu6/tpu6^ line. If that is not able to recapitulate the phenotype, the authors might try knocking down the other differentially expressed genes (BX548011.1 (43-fold decrease in expression) and si:dkey-9c18.3 (29-fold decrease in expression). Alternatively, they might sequence the genome of the mutant94 line to see if it really was inserted in crp2-1, or perhaps was inserted into a master regulator that results in differential crp2-1 expression.

**Major concern: Fig S2.** There a very strong induction of CRP genes in the AB/unchallenged (20 fold?). In particular, the expression of crp2-2 is quite high from the mock challenge, is this a response to KCl or tricaine? There’s data from showing that salt (NaCl) affect leukocyte trafficking and wound healing (see work form Philipp Niethammer and others) so this could be one explanation. Alternatively, does tricaine induce anything? It is surprising that Spn induces crp2-1 instead of 2-2, and given the similarity in these genes, how confident are the authors that these reads are appropriately assigned? Or is there another explanation for this finding?

Line 575: Please describe methods for genotyping (primers and strategy) used to distinguish homozygous / heterozygous / wt. You note its by sanger sequencing but I’m not sure how this was done? Were diseased larvae euthanized at some point before collection? Or checked very frequently before they decompose?

Line 644: Mann Whitney tests are only appropriate for comparison of two experimental groups. Many of the analyses here have more than two groups being compared and a Mann Whitney should not be used: Fig 3, 5. (Kruskal-Wallis, ANOVA, or other tests may be appropriate depending on the comparisons and normality of the samples). No stats are included in Fig 2 or 4, but should be.

The genetic screen used a cross with TL, is it possible differences in this and the AB background lead to your differences / lack of recapitulation in the CRISPR KO of Crp2-1? Why were screens done in TL and targeted deletion in AB? Unless I am misunderstanding the methods here, this is a major concern if the RNAseq is done in TL background but compared to AB?

**Minor Concerns**

Line 72: “one of the most important pathogens in humans” is over stated. Please modify and/or provide a citation for why this is considered to be the case.

Line 76: “respiratory tract infections, such as acute otitis media” – otitis media is not a respiratory tract infection

Line 77-78: It sounds like you are suggesting that otitis media and sinusitis can lead to bacteremia and meningitis? Typically this proceeds from a pneumonia. Please clarify and provide citations either way.

Line 79-80: “Moreover, pneumococcus is responsible for about a half a million child deaths each year making it one of the most common causes of lethal infection at young age” – This statement again seems a bit over stated (and I don’t think its necessary for the authors to over emphasize). The papers cited do not state that claim, and one estimates between 200-300k per year, with a note that it could be as high as 500k (in 2015, and there has been increasing PCV vaccination as of 2020). Other estimates from the WHO seem closer to 300k, so rounding to “half a million” and “the most common cause” is a bit strongly worded.

Line 97-99: This paper also demonstrates a link to complement deficiencies which is relavent to your conclusions. It also states theres not a big link with defects in phagocytosis though (which you go on to emphasize in line 102).

Line 105: NOD should be capitalized (not Nod) as its an abbreviation

Line 109: Citation 25 concludes there is no association with MBL2. Please double check citations to ensure conclusions are properly reported.

Line 110: “As a result, the understanding of the genetic basis of pneumococcal disease remains limited.” The authors suggest this is due to poorly conducted meta analyses (this is what is concluded in citation 25, which the authors build from). An alternative conclusion is that the polymorphisms leading to susceptibility are variable, as might be predicted by the variability in disease outcome, severity, etc seen in clinical infection (humans and is recapitulated in animal models). The authors seem to be suggesting that zebrafish or animal models in general might be a better way to assess this question, but ultimately, the only way to determine what human genes are important is to look in humans. I think the author’s foundation /intro would be much stronger by emphasizing the power of zebrafish and showing that it does in fact correlate with many findings in humans, and is therefore a useful tool, instead of being over critical of human data.

Line 137: “As a result, we found that the innate immune response in pneumococcal infection in zebrafish is well conserved which further validates the use of this model in the evaluation of the host-pathogen interactions during pneumococcal infection”. Well conserved with what? Presumably humans? The authors do not say what they are comparing to or what parts are conserved.

Line 152: “time point of 18 hpi represents the acute stage of the pneumococcal infection in zebrafish larvae” – Can you clarify what you mean by “the acute stage”?

Line 162-164: What percentage of the genome is this? How does this compare to the number of genes altered in mouse or human acute infection?

Line 185: Table 1: What do the commas signify in the fold change? Were there replicates that were 28-fold and 9-fold (for the first line, c3a.2 as an example). Or do the authors mean 28.9-fold (in which case this should be a decimal).

Table 1 and experimental design: the legend states fold change vs uninfected. Were these truly uninfected or mock-infected (PBS control, still received tricaine and a mock injection?)

Line 193: You state the response is well conserved. Please describe how. (what are you comparing to, a septic infection in mouse and blood transcriptome at a similar timepoint? Or peripheral response in humans with invasive infection during hospitalization?) Please include citations for the comparison to support the claim. Complement function is based on protein mostly, not sure how the transcriptional response compares (as is shown here).

Line 204-206: Two homologs of CRP in zebrafish associated with the activation of complement in humans were induced? This should be two sentences, one saying there are 2 zebrafish homologs of CRP that are induced, and another one explaining the function of CRP in human.

Line 208-211: The authors could do more to explain the function of these genes (RNAse and chitinases are not obviously antimicrobial) – this is not obvious how they might function, and since the article is seeking to characterize the innate immune response to Spn, this is an important point.

Line 248: “gene-breaking” may not be widely recognized and would be helpful to define this genetic approach.

Line 252: Inocula is 30-570 CFU but in the legend it says ~300 CFU. Are you only showing the results from the intermediate dose or are these results in figure 3 polled from multiple experiments? If multiple, what was the range of CFU? Particularly for panel B, where we are comparing CFU in larvae, this varies drastically based on inoculum and is important to note.

**Figure 3B.** It would be nice to change the dots to outline (not filled) so you can see how many samples there are. Currently its hard to distinguish overlapping dots, particularly in AB and mutant14

Line 363: Are these mutants more resistant than other lines, (dose increased from 30-570 to 1412-1880). This is anywhere between a 3-fold to 63-fold increase in dose, either is quite significant in most models.

**Methods:**

Larvaculture – please describe how the zebrafish were raised from 0-6dpf (presumably in an incubator with constant darkness?). Please note if light dark cycling was used during early development or during infection studies.

Line 563: What is sequence type 205?

Line 565: please describe brief culture media and methods for Spn.

Line 566: Why were bacteria resuspended in KCl for some experiments and PBS in others?

Line 568: What agar base was used (Columbia agar+ lamb blood, or TSA agar+ lamb blood etc?)

Line 572: A matter of preference but I would call these mock challenged or uninfected (since you did in fact do control injections).

**Supplemental info:**

Table S1, S2, S3, S4, S5, S6 similar comment as table 1. I believe the authors should have a decimal point instead of a comma in the fold-change column.

Fig S1. Please expand the lower axis and add more tickmarks. Somewhere between 0 and 50 fold change is a huge range!

In case the authors are not familiar, this pre-print may be in interest: <https://www.biorxiv.org/content/10.1101/2022.02.10.479878v1>
